# Antibiotic-driven escape of host in a parasite-induced Red Queen dynamics

**DOI:** 10.1098/rsos.180693

**Published:** 2018-09-12

**Authors:** Elizabeth L. Anzia, Jomar F. Rabajante

**Affiliations:** Institute of Mathematical Sciences and Physics, University of the Philippines Los Baños, Laguna, Philippines

**Keywords:** Red Queen hypothesis, coevolution, antibiotic effectiveness, antibiotic resistance, evolutionary parasitology

## Abstract

Winnerless coevolution of hosts and parasites could exhibit Red Queen dynamics, which is characterized by parasite-driven cyclic switching of expressed host phenotypes. We hypothesize that the application of antibiotics to suppress the reproduction of parasites can provide an opportunity for the hosts to escape such winnerless coevolution. Here, we formulate a minimal mathematical model of host–parasite interaction involving multiple host phenotypes that are targeted by adapting parasites. Our model predicts the levels of antibiotic effectiveness that can steer the parasite-driven cyclic switching of host phenotypes (oscillations) to a stable equilibrium of host survival. Our simulations show that uninterrupted application of antibiotic with high-level effectiveness (greater than 85%) is needed to escape the Red Queen dynamics. Interrupted and low level of antibiotic effectiveness are indeed useless to stop host–parasite coevolution. This study can be a guide in designing good practices and protocols to minimize the risk of further progression of parasitic infections.

## 1. Introduction

Understanding antagonism-mediated evolution is essential as antagonistic interactions, including parasitism, play major roles in the formation and maintenance of the structure of communities [1–5]. The Red Queen hypothesis is a model for winnerless antagonistic coevolution between interacting species, such as host–parasite, prey–predator and victim–exploiter [6–8]. The Red Queen hypothesis has been demonstrated using various schemes, e.g. to explain the evolution of sex [9–11] and the antagonism-mediated species diversity [6,12,13]. Here, we focus on fluctuating Red Queen mode (in contrast to escalatory Red Queen and chase Red Queen [9,14]) to explain the effect of inhibiting the growth of parasites on host–parasite coevolution. One of the numerical manifestations of fluctuating Red Queen mode is the canonical Red Queen dynamics/cycles [15–17].

**Figure 1.**
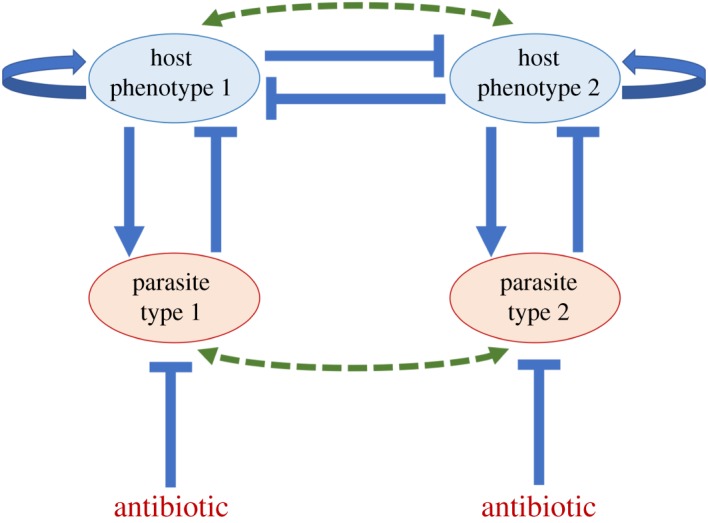
The host phenotype decision-switch network with the influence of parasitism. Self-regulation in hosts means host growth and phenotypic memory. Host phenotypes mutually inhibit each other to characterize trait selection, but simulations can show coexistence is possible. Parasites proliferate through infection but their growth can be suppressed by antibiotics. Solid arrows denote positive interaction. Solid bars denote negative interaction. Dashed arrows imply evolution.

**Figure 2.**
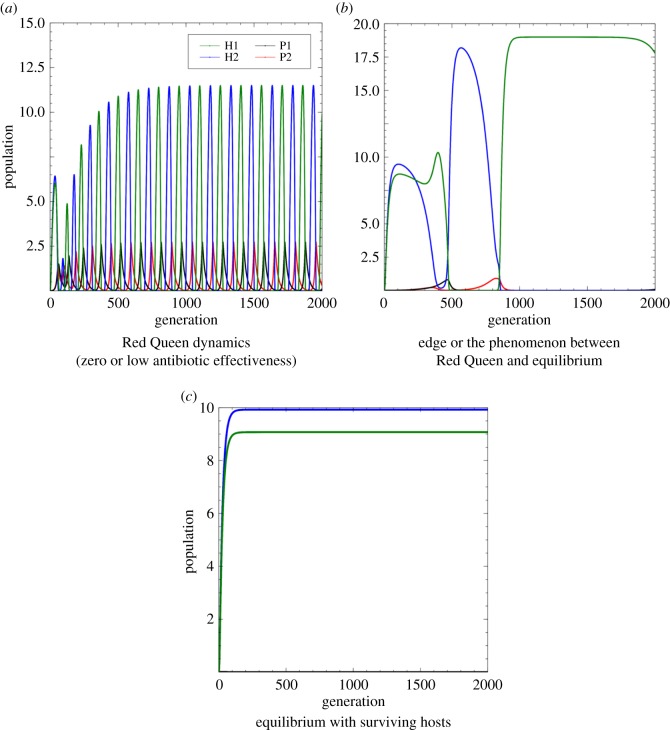
Different qualitative behaviours of host–parasite interaction with the additional effect of antibiotics to the parasite. (*a*) Oscillating population abundance of hosts and parasites as they interact with each other. The effect of insufficient levels of antibiotic to eradicate parasitism can also result in Red Queen dynamics. (*b*) Irregular oscillating population abundance of hosts and parasites due to the insufficient effect of antibiotic. (*c*) The population abundances of hosts and parasites converge to an equilibrium because the levels of the antibiotic are sufficient to suppress parasitism.

The Red Queen dynamics in host–parasite system describes winnerless coevolution between hosts and parasites, realized through perpetual negative frequency-dependent selection [16]. The canonical case of the Red Queen dynamics follows the following process: common host population (say, H1) evolves to a new common type (H2) to escape their parasites (P1). However, the decline in the population of H1 will drive parasite population P1 to evolve into a new common strain (P2) as a response against the evolution of the host. The new common parasite P2 can infect H2, which will drive the hosts to evolve again, resulting in never-ending alternating cycles of dominance (figures 1 and 2*a*). For example, the Red Queen dynamics explain coevolution in invertebrate–parasite systems, such as infection of *Daphnia magna* by bacteria *Pasteuria ramosa* [18,19].

The canonical Red Queen dynamics can be characterized by populations of host types (and parasite types) undergoing cyclic dominance switching in population abundance, where every host (parasite) type has the opportunity to have an abundant frequency for some period of time [16]. Mathematically, we define Red Queen dynamics as population oscillations that are out-of-phase with similar amplitude, usually heteroclinic [6,16]. This definition is consistent with the Red Queen hypothesis inspired by Lewis Carroll's *Through The Looking Glass*: ‘it takes all the running you can do’ (dominance switching represented by out-of-phase cycles), ‘to keep in the same place’ (oscillations with similar amplitude, representing host fitness remaining the same even though there is coevolution) [16,20]. Moreover, one of the properties of the canonical Red Queen dynamics is that the population sizes of a host type can reach its maximum near the value of the carrying capacity, but with the associated risk of extinction (impermanent coexistence) when negative-frequency selection occurs in favour of other host types [6,16,21,22].

The Red Queen dynamics is akin to the kill-the-winner hypothesis in bacteria–phage systems [23,24]. The fitness of a common host type decreases due to parasitism, initiating the escalation in the fitness of a rare host type. The new common host type will then be the target of parasitism [6]. When hosts fail to survive the coevolution, then their population may converge to extinction. Similarly, parasites that fail to catch up with the evolving hosts may soon be eradicated [9]. To simulate the Red Queen dynamics with the perpetual negative frequency-dependent selection, we assume that the persistence of Red Queen dynamics materializes in conditions where hosts and parasite can avoid extinction and recover when they reach very low density [16]. The parameter range leading to the Red Queen dynamics is wide in deterministic host–parasite systems because there is symmetrical selection, that is, host evolution is countered by the evolution of parasites, especially in the absence of alternative hosts [6,9]. The factors that foster the Red Queen cycles are strong repression to express multiple host types (inter-host type competition), adequate basal host birth rate for survival, sufficient carrying capacity of the host's environment, high degree of parasite specificity (e.g. matching-allele interactions) and intermediate levels of parasite mortality [16]. Inter-host type competition characterizes evolutionary selection among host types (genotype or phenotype) that will be commonly expressed in the host population. In multi-type host–parasite systems, multiple host types may coexist, but if canonical Red Queen dynamics arises, only one host type is common/dominant for a certain period of time [25].

Intermediate level of parasite mortality is essential in maintaining Red Queen dynamics. Low parasite death rate imposes a high degree of parasitism that adversely affects the host populations; while high parasite death rate limits the antagonistic influence of parasites to initiate negative frequency-dependent selection in hosts [16]. If it is desired to escape the coevolution in Red Queen dynamics, then we can do various strategies, such as suppressing the parasitic functional response in host population (e.g. through introduction of probiotics), suppressing the numerical response in parasite population (e.g. through introduction of antibiotics) and increasing death rate of parasites (e.g. through introduction of a different kind of antibiotics). Here, ‘escape’ means stopping the parasitism-driven Red Queen cycles, where the outcome is a surviving stable host population. We focus on the mathematical investigation of the effect of suppressing the numerical response in parasite population through the introduction of antibiotic to attain a stable positive host population abundance. The inhibiting factor that suppresses the growth of parasites is referred to as *antibiotic*; although, our results can be applied beyond bacterial parasites but without side effect to the host.

Our minimal mathematical model, for the first time, explains a strategy for escaping the Red Queen dynamics via application of antibiotic to reduce reproduction of parasites at the population level. We considered the conditions favourable in steering the Red Queen dynamics as the starting point of our simulations. Then we show the levels of antibiotic effectivity that can stop the cycles. We also show if the interrupted or discontinued application of antibiotic will provide an opportunity for the parasites to recover. Our model assumes that hosts are killed or castrated once infected by the parasite, that is, they cannot recover from disease nor further reproduce. This is often the case in ecological systems with castrating parasites [26–28].

## 2. Mathematical model

Mathematical modelling is very useful in understanding host–parasite interaction and diseases [6,29–31]. The phenotype decision-switch network in figure 1 is used to illustrate the interaction among host phenotypes and parasites and the effect of antibiotic [25]. Here, the expression of host phenotype 1 (H1) inhibits the expression of host phenotype 2 (H2), and vice versa. Moreover, parasites decrease the frequency of the phenotype they are attacking due to infection. As indicated in the network (figure 1), parasitism has high specificity, where parasite 1 (P1) and parasite 2 (P2) target H1 and H2, respectively. We assume that there is a differential effect of antibiotic to each parasite, and the antibiotic does not have side effect to any of the hosts. In our simulations, the mathematical model of host–parasite interaction with an antibiotic is as follows:

2.1 $$\frac{dH_{1}}{dt}=\overset{\mathrm{hostgrowth}}{\overbrace{\frac{r_{1}}{H_{1}+\gamma_{12}H_{2}+1}\;H_{1}}}-\overset{\mathrm{hostmortality}}{\overset{}{\overbrace{\rho_{1}H_{1}}}}-\overset{\mathrm{functional response}}{\overset{}{\overbrace{\frac{\alpha_{1}P_{1}}{1+H_{1}}H_{1}}},}$$

2.2 $$\frac{\text{d}H_{2}}{\text{d}t}=\frac{r_{2}}{H_{2}+\gamma_{21}H_{1}+1}H_{2}-\rho_{2}H_{2}-\frac{\alpha_{2}P_{2}}{1+H_{2}}H_{2},$$

2.3 $$\frac{\text{d}P_{1}}{\text{d}t}=-\overset{\text{parasite mortality}}{\overbrace{d_{1}P_{1}}}+\overset{\text{parasite growth}}{\overbrace{\frac{\xi_{1}H_{1}P_{1}}{1+H_{1}}}}\overset{\;\text{effect of antibiotic}}{\overbrace{e^{-\delta_{1}}}}$$

2.4 $$\;\text{and}\;\frac{\text{d}P_{2}}{\text{d}t}=-d_{2}P_{2}+\frac{\xi_{2}H_{2}P_{2}}{1+H_{2}}e^{-\delta_{2}}.$$

Two existing host and parasite types are considered in this study. To highlight the interaction of host and parasite with the effect of antibiotic, certain simplifying assumptions are considered. First, the hosts have similar characteristics, that is, the growth coefficients of H1 and H2 are equal (*r*_1_ = *r*_2_ = *r*), and they have equal death rates (*ρ*_1_ = *ρ*_2_ = *ρ*). Both the parasite growth and death rates are also equal, that is, *ξ*_1_ = *ξ*_2_ = *ξ* and *d*_1_ = *d*_2_ = *d*, respectively. Furthermore, parasitism efficiency is the same for both parasites (*α*_1_ = *α*_2_ = *α*), and effects of antibiotic to both parasites are equal (*δ*_1_ = *δ*_2_ = *δ*). Lastly, the strength of competition for both H1 and H2 is equal to 1 (*γ*_12_ = *γ*_21_=1), meaning both hosts have similar competitive abilities. These simplifying assumptions characterize a host–parasite system where the hosts are from the same family of species, and the parasites are of closely similar types except that each parasite strain targets different host phenotype. The specificity of parasites characterizes a system where the hosts are able to evolve or adapt against the infecting parasite strain. Table 1 summarizes the parameters used in the model and their description.

**Table 1.** Table of parameters. All parameters are non-negative.

parameter	definition
*r*_*i*_	growth coefficient of host *i*
*ρ*_*i*_	death rate of host *i*
*ξ*_*i*_	numerical response coefficient of parasite growth *i*
*d*_*i*_	death rate of parasite *i* not due to antibiotic
*α*_*i*_	parasitism efficiency of parasite *i* in infecting host *i*
*γ*_*ij*_	coefficient associated with the inhibition of host phenotype *i* by phenotype *j* ≠ *i*
*δ*_*i*_	level of effectiveness of antibiotic targeting the growth rate of parasite *i*

**Figure 3.**
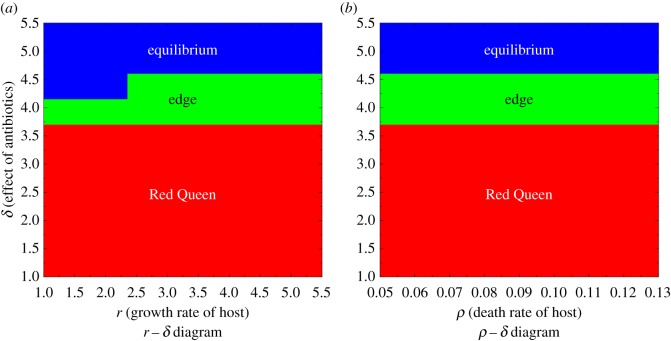
Parameter diagrams showing the qualitative behaviour of host populations when varying different phenotypic properties and the level of effectiveness of the antibiotic *δ*. Parameters *d* = 0.05 and *ξ* = 5 are fixed. (*a*) Varying growth coefficients of hosts (*r*) versus effectiveness of antibiotic (*δ*); *ρ* = 0.12. (*b*) Different death coefficients of hosts (*ρ*) versus effectiveness of the antibiotic (*δ*); *r* = 4.

**Figure 4.**
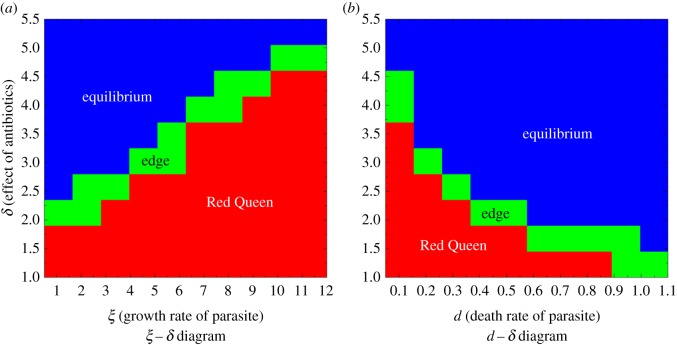
Parameter diagrams showing the qualitative behaviour of host populations when varying different parasite characteristics and the level of effectiveness of the antibiotic *δ*. Parameters *r* = 4 and *ρ* = 0.08 are fixed. (*a*) Parasite growth rates (*ξ*) versus different levels of effectiveness of antibiotic (*δ*); *d* = 0.05. (*b*) Death rates of parasites (*d*) versus different levels of effectiveness of antibiotic (*δ*); *ξ* = 5.

**Figure 5.**
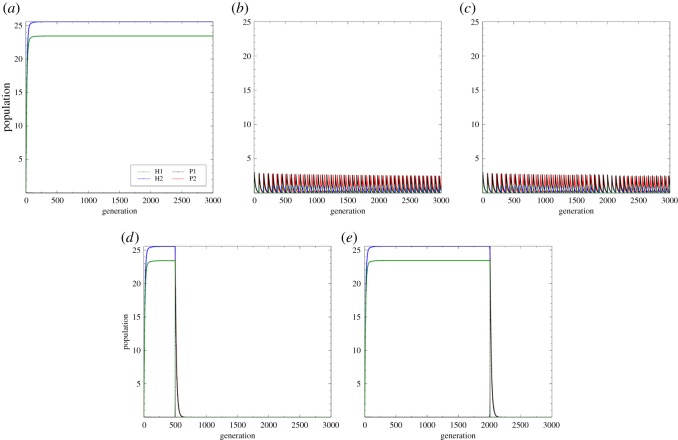
Different modes of the antibiotic application resulting in different population dynamics. (*a*) Equilibrium convergence showing the coexistence of the hosts and the full suppression of parasitism because of the uninterrupted application of antibiotic. (*b*) Oscillating population of the hosts and parasites but with very low amplitude; the application of antibiotic is every 1 simulation period. (*c*) Oscillating population of the hosts and parasites but with very low amplitude; the application of antibiotic is every 30 simulation periods. (*d*) Equilibrium dynamics with a sudden decline in host population abundance when the antibiotic was applied continuously but is discontinued after 500 simulation periods. (*e*) Equilibrium dynamics with a sudden decline in host population abundance when the antibiotic was applied continuously but is discontinued after 2000 simulation periods.

The combinations of parameter values in the simulations (*r*, *ρ*, *ξ* and *d*) conform to the Red Queen dynamics in host–parasite system with Type II functional response [16]. The parameter values presented in table 2 are used in the simulations (100 000 simulation runs) shown in figures 2–5. Other combinations leading to the Red Queen dynamics outside the parameter ranges in table 2 result in similar conclusions. The tested levels of effectiveness of the antibiotic are presented in table 3. The level of effectiveness is a function of the amount of antibiotic (*Y*) and efficacy (*ϕ*). Without losing generality, we set *δ* = *ϕY* . Effectiveness of antibiotic to fully suppress parasite population growth depends on the value of *ξ*. The higher the *ξ*, the higher is the *δ* possibly needed. With the addition of an antibiotic, we inspect if the host–parasite coevolution will escape the Red Queen dynamics, and how much *δ* is needed to realize this.

**Table 2.** Table of parameter values used in the simulations. L, ML, M, MH and H respectively mean low, medium low, medium, medium high and high values in relation to the parameter range of the Red Queen dynamics.

parameter	L-a	L-b	ML-a	ML-b	M-a	M-b	MH-a	MH-b	H-a	H-b
*r*	1	1.5	2	2.5	3	3.5	4	4.5	5	5.5
*ρ*	0.05	0.055	0.06	0.07	0.08	0.09	0.1	0.11	0.12	0.13
*ξ*	0.5	0.75	1	1.5	2	3.5	5	7.5	10	12
*d*	0.05	0.15	0.3	0.4	0.5	0.65	0.8	0.9	0.99	1

**Table 3.** Tested level of effectiveness of the antibiotic.

*δ*	e^−*δ*^	effectiveness = 1 − e^−*δ*^
1	0.368	0.632
1.5	0.223	0.777
2	0.135	0.865
2.5	0.082	0.918
3	0.050	0.950
3.5	0.030	0.970
4	0.018	0.982
4.5	0.011	0.989
5	0.007	0.993
5.5	0.004	0.996

Uninterrupted antibiotic application means that the antibiotic is replenished before its effect declines. Here, we also simulate cases where the antibiotic application is interrupted (periodic) and discontinued. We denote *τ* as the gap period between antibiotic applications. For example, *τ* = 30 means that antibiotic is applied to reduce parasite population growth every after 30 simulation periods. During the gaps, the antibiotic has no effect. The model that we use to reflect the implication of gaps between antibiotic applications to the parasite population growth (*i* = 1, 2) is

2.5 $$\frac{\text{d}P_{i}}{\text{d}t}=-d_{i}P_{i}+\frac{\xi_{i}H_{i}P_{i}}{1+H_{i}}\;{\text{e}}^{-\delta \lfloor 0.50001(\sin(2\pi t/\tau )+1)2+0.5\rfloor }.$$

## 3. Results and discussion

The existence of Red Queen dynamics in host–parasite interaction follows from the existence of parasites, and eradication of parasites can lead to the escape from Red Queen dynamics. We started with an initial state where the host–parasite interaction exhibits Red Queen dynamics at the population level. Then we answer the question: what level of antibiotic effectiveness can stop the Red Queen cycles leading to the stable survival of one or more host types? We intend to escape the Red Queen dynamics for two main reasons: (i) to eliminate or minimize parasite infection leading to the winnerless coevolution of hosts and parasites and (ii) to avoid the risk of extinction of hosts (impermanent coexistence), especially when there is demographic stochastic noise in host population growth [32]. The possibility of impermanent coexistence in hosts could make the Red Queen dynamics hardly observable in nature [33,34].

Based on our simulations, the host–parasite interaction still exhibits Red Queen dynamics (figure 2*a*) even with the introduction of antibiotic but with low effectiveness. This suggests that the level of antibiotic is inadequate to eradicate parasitism. In the presence of an ineffective antibiotic, cyclic host population abundance can still have high amplitude similar to the host–parasite system without antibiotics (figure 2*a*). One of the positive consequences of this ineffective antibiotic is that it is possible that the time period before reaching impermanent coexistence widens. However, still the hosts are not able to escape the Red Queen dynamics, with impermanent coexistence as a possible associated recurring risk.

Increasing the level of effectiveness of the antibiotic may lead to a phenomenon wherein the oscillations become irregular (referred to as the edge between the Red Queen dynamics and equilibrium; figure 2*b*). This happens when the antibiotic has partial effect on the parasite, driving a decrease in parasite efficiency but only for a limited duration. To totally suppress parasitism and escape the Red Queen dynamics, a greater level of effectiveness of the antibiotic is needed (figure 2*c*). The suppression of parasitism leads the hosts to a state of equilibrium wherein one or both host types converge to have non-zero population sizes. As shown in figure 2*c*, both the host types may coexist; however, a specific host could be more dominant than the other host.

As illustrated in figure 3, the host needs the level of effectiveness of antibiotic to be *δ* > 4 or *δ* > 4.5 in order to escape the Red Queen. Based on table 3, this indicates that the antibiotic should be greater than 98.2% or greater than 98.9% effective to destroy parasites, which is close to an ideal antibiotic. A counterintuitive result shows that a more effective antibiotic (greater than 98.9%) is needed to escape the Red Queen dynamics when host growth rate is higher (comparing *δ* associated with *r* < 2.5 and *r* > 2.5 in figure 3*a*). This is because a high host growth rate is favourable to generate Red Queen cycles [16].

We investigate the relationship between the parasite growth and death rate, and the effectiveness of the antibiotic. As shown in figure 4*a*, the growth rate of the parasite (*ξ*) has a direct relationship with the effectiveness of the antibiotic needed to escape the Red Queen dynamics. The higher the parasite growth rate, the greater the effectiveness of the antibiotic is needed. This is logical since the eradication of parasites is more difficult if they have a high growth rate. The parasite with less growth rate (*δ* = 1) will be suppressed using a 91.8% effective antibiotic. However, the parasite with high growth rate (*δ* = 10) will be suppressed using 99.6% effective antibiotic. On the other hand, the parasite death rate (*ρ*) has an inverse relationship with the effectiveness of the antibiotic (figure 4*b*). As the death rate of the parasite increases, the lower the antibiotic effectiveness is needed to escape the Red Queen dynamics. This is because high parasite mortality (not due to antibiotic) can hasten the parasite's extermination when antibiotic is introduced. A parasite death rate of *d* = 1 will result in full suppression of parasitism if we introduce 86.5% effective antibiotic.

Our simulations show that uninterrupted application of effective antibiotic could lead to the full suppression of parasitism through the reduction in parasite population growth. However, this result may change when we vary the application periods of antibiotic (i.e. on specific simulation periods only). We investigate the dynamics of the hosts and parasites using the extended model in equation (2.3) with varying values of the parameter *τ* with other parameters being fixed. Compared to the case where the antibiotic application is uninterrupted (figure 5*a*), the abundances of both host and parasite populations are low when antibiotic is applied periodically (figure 5*b*,*c*). Moreover, the periodic application of the antibiotic may lead to oscillatory population sizes, with the population dynamics of the hosts having a very low amplitude (figure 5*b*,*c*). This interrupted antibiotic application is more adverse to the hosts compared when the antibiotic is less effective but applied continuously. This suggests that unsustainable antibiotic application could result in more potent parasite infection. In figure 5*d*,*e*, we show that discontinuing the application of antibiotic, even for a short time, may result in the death of hosts. The parasites could become more potent that drives host extinction. This is worse compared to the Red Queen dynamics without antibiotics, since host populations undergoing Red Queen cycles still have the opportunity to survive through deterministic coevolution. Uninterrupted application of antibiotic is recommended until parasite population is completely eradicated or, possibly, until a level where host immunity can inhibit parasite infection.

## 4. Conclusion

Using mathematical simulations, we show two novel testable hypotheses: (i) for the hosts to escape the Red Queen dynamics, a high level of antibiotic effectiveness is needed and (ii) interrupted or discontinued application of antibiotic could be detrimental to the hosts. Here, the introduced antibiotic suppresses the population growth of the parasites which will then result in parasite eradication. However, if the application of the antibiotic is discontinued, surviving parasites can recover and these parasites can be more potent causing the sudden extinction of hosts. This suggests that for the hosts, the case where antibiotic is introduced but cannot be sustained is riskier compared to the case of Red Queen dynamics without antibiotic. Red Queen dynamics can drive impermanent coexistence in hosts but the hosts have the chance to survive through deterministic cyclic coevolution. Thus, the use of antibiotic should be regulated ensuring its effectiveness even though costly.

## Data accessibility

Code is available from the Dryad Digital Repository: https://doi.org/10.5061/dryad.3c7pd3g [35].
